# Modular CBT for Childhood Anxiety Disorders: Evaluating Clinical Outcomes and its Predictors

**DOI:** 10.1007/s10578-022-01437-1

**Published:** 2022-10-03

**Authors:** Francisca J. A. van Steensel, Liesbeth G. E. Telman, M. Maric, Susan M. Bögels

**Affiliations:** 1 Research Institute of Child Development and Education, University of Amsterdam, Nieuwe Achtergracht 127, Postbus 15776, 1001 NG, 1018 WS Amsterdam, The Netherlands; 2 Department of Developmental Psychology, University of Amsterdam, Nieuwe Achtergracht 127, 1018 WS Amsterdam, The Netherlands; 3 Department of Clinical Child and Family Studies, Utrecht University, P.O. Box 80140, TC 3508 Utrecht, The Netherlands

**Keywords:** Anxiety disorders, Children, Modular CBT, Effectiveness, Predictors

## Abstract

This study examined clinical outcomes of a modular individual CBT for children with anxiety disorders (AD), and predictors of outcomes, in usual clinical practice. Participants were 106 children with ADs (7–17 years), and parents. Assessments were pre-, mid-, post-test, and 10 weeks after CBT (follow-up). Predictors (measured pre-treatment) were child characteristics (gender, age, type of AD, comorbid disorders), fathers’ and mothers’ anxious/depressive symptoms, and parental involvement (based on parents’ presence during treatment sessions and the use of a parent module in treatment). At follow-up, 59% (intent-to-treat analyses) to 70% (completer analysis) of the children were free from their primary anxiety disorder. A significant decrease in anxiety symptoms was found. Higher parental involvement was related to lower child anxiety at follow-up, but only for children with comorbid disorders. Findings suggest that it is beneficial to treat anxiety with modular CBT. Future steps involve comparisons of modularized CBT with control conditions.

**Supplementary Information:**

The online version contains supplementary material available at 10.1007/s10578-022-01437-1.

## Introduction

Almost three decades ago, Weisz and colleagues [1] made a distinction between ‘research therapy studies’ and ‘clinic therapy studies’. Research therapy studies include participants who have been recruited and selected (following in- and exclusion criteria) for a particular study; treatment is given by trained and supervised (research) staff, and is highly structured with a fixed number of sessions and close monitoring of treatment adherence. In contrast, clinic therapy studies include participants who have been referred for their problems (which are often more severe, heterogeneous, and require a more broad multiproblem focus) and who are treated by clinicians who have large (and diverse) caseloads, and who may use a multimodal, eclectic, or more flexible approach (instead of following a treatment protocol). Similar to the distinction between ‘research therapy studies’ and ‘clinic therapy studies’, a distinction is made between efficacy studies (results of an intervention under ideal and controlled circumstances) and effectiveness studies (results of an intervention under ‘real world conditions’) [2]. Efficacy studies will have high internal validity, while effectiveness studies will have high external validity. However, often studies can not be categorized as either an efficacy or an effectiveness study; i.e., studies have characteristics of both (efficacy and effectiveness), and therefore may be placed somewhere on the efficacy-effectiveness continuum [2]. The current study may be placed more on the effectiveness side of the continuum, as its aim was to examine treatment outcomes and predictors of change, for children with anxiety disorders, using a clinically representative sample and a more flexible treatment approach.

Cognitive Behavioral Therapy (CBT) is effective for treating anxiety disorders in children (e.g., effect size of CBT compared to no therapy: *d* = 0.76; [3]). However, about one third of children are not free from their primary anxiety disorder after treatment [4]. Most of the previous (research therapy) studies used a standard treatment protocol with the same session outline for all children and a fixed number of sessions. However, in clinical practice, therapists often adjust these treatment protocols to the needs of an individual child, for example by choosing parts of the protocol or adjusting the number of sessions. Although this approach may be understandable considering high heterogeneity found in clinical practice, we are in need of methods that could bridge this gap between research therapy studies and clinical practice. One way, gaining in popularity, is to use a modular treatment format. Modular CBT is an innovative way of adapting an intervention to the individual, by dividing an evidence-based treatment into self-contained modules [5]. In this way, modular CBT provides a structured approach while at the same time the therapist is able to choose which modules to use, and in what order. Therefore, treatment of two different children with the same manual may look different because of the chosen modules or the order of the modules. Support for the use of modular treatment comes from a study [6] that included 174 youths with anxiety, depression, trauma, and/or conduct problems who received intervention for their problems and where therapists were randomized by providing either modular treatment, standard evidence-based care (following a protocol), or usual care. The modular format outperformed both usual care and standard evidence-based treatments [6, 7]. The authors suggested that this was due to a more flexible approach as they reported that in the modular condition therapists used more evidence-based content than in usual care condition, and that therapists used more ‘other’ content than in the standard evidence-based care condition. A limitation to that study was that sample sizes were too small to conduct separate analyses for the specific target groups (e.g., children with anxiety disorders). Further evidence for the effectiveness of a modular CBT for anxiety disorders comes from studies that have applied modular CBT in school-based settings. Those studies did not compare a modular format to a comparison condition, but did report positive results in terms of treatment effectiveness (decrease in anxiety symptoms) and treatment response (free of anxiety disorder, clinically improved) [8–10].

Previous studies (using non-modular CBT) have examined the role of several child- and parent predictors on treatment effectiveness. Reviews and meta-analyses suggest that child gender or age did not predict treatment outcome [3, 11], but other child pre-treatment characteristics such as type of AD or comorbidity have been found to predict treatment outcome. Several studies indicated that having a diagnosis of social anxiety disorder (SAD) is a predictor of less favorable outcomes compared to children with generalized anxiety disorder (GAD), separation anxiety disorder or specific phobias [12, 13]. In addition, comorbid depressive symptoms were found to be a predictor of less favorable CBT outcomes for children with anxiety disorders [12, 14], whereas no negative treatment effect was found for hyperactivity symptoms [15] or autism spectrum traits [16, 17].

Next to child factors, parents’ own anxiety symptoms/disorders and parental involvement have been associated with treatment effectiveness. However, for both factors evidence is inconsistent. That is, a study [18] found parents’ own anxiety (disorders) to be associated with worse treatment outcome, while other studies did not find a relation between parental anxiety and treatment outcome [19, 20]. In this regard, some studies point to the relevance of studying fathers’ and mothers’ anxiety separately. For example, a study [21] found that maternal (but not paternal) anxiety symptoms were associated with better treatment outcome in adolescents. However, in contrast, it has also been found that fathers’ (but not mothers’) anxiety symptoms were predictive of worse treatment outcome [22, 23].

With respect to parental involvement, there is large heterogeneity in the way and extent to which parents are involved in children’s anxiety treatment: e.g., parents can be present only once or twice in sessions, parents may have parallel sessions, or parents (and other family members) may be present during all sessions; parents may be involved as co-therapists and/or may be learnt how their parenting behaviors (modeling, overprotection, autonomy granting) may be related to their child’s anxiety, and/or may be involved (only) in contingency management [24, 25]. Overall, (meta-analytical) reviews [3, 26–28] comparing child CBT to family CBT (CBT with parental involvement) do not find support that family CBT is more effective than child CBT in the treatment of childhood anxiety disorders. However, there is also evidence that specific parental treatment components (i.e., contingency management and/or transfer of control) are beneficial for children’s *long-term* anxiety remission rates, and that high parental involvement (expressed as parents being present in all sessions or receiving the same number of sessions as their child) is beneficial for *long-term* secondary treatment outcomes (e.g., depressive symptoms, social competence) [24, 25]. In the current study, parental involvement was operationalized as (1) the presence of the parent in the treatment sessions, and (2) the use (yes/no) of ‘parental content’ in treatment (i.e., parenting style, parents’ own dysfunctional cognitions, coparenting, and communication about anxiety).

Despite the heterogeneity in parental involvement in previous research, it may be that some child characteristics interact with parental involvement. For example, previous research found that family CBT—in which parents were trained to reward courageous behaviors of the child, to manage their own emotions and use modelling, and to train their communication and problem solving skills—was more effective than child CBT for younger children compared to older children [29]. This finding may be related to the developmental phase of adolescents in which support by friends is increasing while parental support is decreasing [30]. Another child characteristic that may interfere with parental involvement is comorbidity. For example, for children with high attention deficit problems family CBT was found to be more beneficial than child CBT [31], and similar results were found for children with high autistic traits [16]. It might be that children with comorbid problems (e.g., ASD) are more dependent on their parents to apply learnt skills in daily settings and that parents need to be involved in treatment in order to generalize (in the long term) what is learnt in treatment [32], and/or that children with comorbid problems (e.g., ADHD) need more parental involvement in treatment to make sure that they complete homework assignments.

In the current study, our first aim was to examine the clinical outcomes of a modular CBT in a sample of children who were referred to several community mental health care clinics. Following the results of previous studies [6–10], we hypothesized that anxiety would decrease after having followed modular CBT. The second aim was to investigate child and parent predictors (e.g., child gender, child age, type of anxiety disorder of the child, parental anxiety/depression, and parental involvement) of change in anxiety. As previous studies examining predictors were all studies that have used non-modular (standardized/protocolized) CBT, and we do not know whether predictors may act differently for modular CBT, this second aim was more explorative. We did not expect that gender and age would predict treatment outcomes per se, as previous studies did not find much evidence for gender or age to be related to treatment effectiveness [3, 11] and we could not reason why this would be different for modular CBT. Previous studies did find support for a more negative treatment outcome when children had a social anxiety disorder [12, 13]. However, modular CBT might be able to give the therapists the flexibility to use more time for certain aspects of the treatment that might be beneficial for children with social anxiety disorder (i.e., targeting self-directed attention [33] or additional problem solving sessions [34], or to add additional sessions). Considering the inconsistent results for parents’ own anxiety/depression symptoms and parental involvement (see above), we did not formulate explicit hypotheses regarding these predictors. Our third aim was to examine whether the effect of parental involvement on changes in anxiety symptoms was associated with child age or child comorbidity. We expected that parental involvement would interact with child comorbidity and child age in a way that parental involvement is beneficial for children with comorbidity (based on the results of previous studies [16, 31]) and for younger children (based on a previous study [29] and given the developmental phase of adolescents [32]).

## Material and Methods

### Participants

Participants were 106 children referred with anxiety disorders (53 boys; 50.0%) aged 7–17 years (mean age = 11.04, SD = 2.44), and their parents. Of the parents, 99 mothers (93.4%) and 79 fathers (74.5%) participated. Their mean age was 43.83 (SD = 4.88) and 46.23 (SD = 5.42) respectively. Parents filled in their highest educational level which was divided in five categories: (1) primary school (0% of the mothers/1.5% of the fathers) (2) secondary school (8.8% of the mothers/13.2% of the fathers), (3) middle level vocational school (35.2% of the mothers/22.1% of the fathers), (4) bachelor degree (24.2% of the mothers/36.8% of the fathers), and (5) master degree or higher (28.6% of the mothers/25.0% of the fathers).

Of the 106 children referred to one of the mental health care centers for anxiety problems, 96 were administered a semi-structured clinical interview for DSM-5 disorders for children (SCID-junior [35]) at pre-test to establish the presence of DSM-5 (anxiety) disorders (n = 10 did not complete the interview assessment prior to treatment, but did have a clinical DSM-5 anxiety disorder, and completed the questionnaires at pre-treatment assessment). All 96 children were found to have at least one anxiety disorder; most common being generalized anxiety disorder, social anxiety disorder and specific phobia (see Table 1). Most children had more than one anxiety disorder (mean number of anxiety disorders = 2.01, SD = 0.98; 60 children (56.6%) had a comorbid anxiety disorder. Although 55 children (51.9%) had a comorbid disorder other than an anxiety disorder (see Table 1), the principal diagnosis to be treated was the anxiety disorder. Thirteen children were using medication (11 for ADHD-related problems and two for anxiety/mood related problems) which was kept stable during the study trial. In addition, 20 families received additional treatment next to or after CBT but this treatment was not targeting anxiety problems; 11 parents received psycho-education for their child’s other problems (e.g., ADHD/ASD), 6 families received guidance in the home setting (e.g., help for planning daily activities with schedules and/or pictograms, or for the child to attend supervised free-time activities). Further, around the time of the follow-up measurement (10 weeks after following CBT for anxiety problems) one child started social skills training, one child started mindfulness training, and one child followed a self-esteem training.

**Table 1:** Overview of the types of anxiety disorders and comorbid disorders based on the semi-structured interview for DSM-5 disorder (SCID-junior)

	N = 96*	%
*Primary anxiety disorder (AD)*		
Generalized anxiety disorder	31	32.3
Social anxiety disorder	30	31.3
Specific phobia	22	22.9
Separation anxiety disorder	11	11.5
Panic disorder	1	1.0
Selective Mutism	1	1.0
*Comorbid anxiety disorder*	**60**	**56.6**
Generalized anxiety disorder	29	30.2
Social anxiety disorder	22	22.9
Specific phobia	26	27.1
Separation anxiety disorder	14	14.6
Panic disorder	5	5.2
Agoraphobia	1	1.0
*Comorbid disorder (not AD)*	**55**	**51.9**
Attention Deficit Hyperactivity Disorder	30	31.3
Insomnia	23	24.0
Mood Disorder	9	9.4
Obsessive Compulsive Disorder	7	7.3
Autism Spectrum Disorder	3	3.1
Illness Anxiety Disorder	3	3.1
Post-Traumatic Stress Disorder	3	3.1
Oppositional Defiant Disorder	3	3.1
Disruptive Mood Dysregulation Disorder	1	1.0

*SCID-junior assessment was missing at pre-treatment for 10 cases

### Procedure

Mental health care centers and therapists were asked to participate in the study. The mental health care centers were typical community centers not specifically specialized in treating anxiety disorders. No in- or exclusion criteria were set for a mental health care center or therapist to join the study. In addition, no in- or exclusion criteria were set for the participants other than (1) having an anxiety disorder, (2) having at least one parent who wanted to participate in the research, and (3) the therapist/multidisciplinary team indicated anxiety treatment. Thus, children with anxiety problems were referred to one of the 20 participating mental health care centers and were asked to participate in the study when the multi-disciplinary team decided that anxiety treatment was needed. When families agreed to participate, the researcher was informed and families were contacted for their first assessment. Informed consent was obtained from all individual participants (aged 12 years and older) and from their parents, and Ethical approval for the study was given by the ethical committee of the university. There were four assessments: before treatment started (pre), after 5 sessions (mid), directly after treatment (post), and 10 weeks after treatment (follow-up). Families received a 20 euros gift card after they finished all assessments. Assessments (including the administration of the semi-structured interview) were conducted by the second author and research assistants (master students under the supervision of the second author). The second author and research assistants were trained in the assessment of the SCID-junior, and were independent from the clinicians who treated the children.

### Intervention

All children received a modular CBT to treat their anxiety problems. This modular CBT is an adapted version of the protocol Discussing + Daring = Doing [36] which consists of 12 child sessions and 3 parent sessions and is proven effective to treat anxiety disorders in children [17, 18]. The adapted modular CBT consists of the following modules: (1) Psycho-education, (2) Cope (including task concentration), (3) Think (cognitive restructuring), (4) Feel (relaxation + mindfulness), (5) Do (experiments + exposure), (6) Parental guidance (i.e., parenting style, parents’ own dysfunctional cognitions, coparenting, and communication about anxiety), and (7) Relapse prevention. In this study, the modules Psycho-education and Relapse prevention were compulsory in respectively the first and last session. For the remaining sessions, therapists (together with clients) were free to choose any module which they thought (based on intake, pre-treatment information, and clinical judgement) would be helpful to overcome the child’s anxiety problems. In addition, therapists (together with clients) decided on the number of the sessions, as well as on homework assignments and parental involvement. This way, therapists could adjust the amount of treatment content in a session. For example, if a child needed more time to go through the exercises of a module, a therapist could spent 2 or 3 sessions on that same module (or repeat the same module).

The children in this study were treated by 67 therapists (n = 62 women; 92.5%). On average they had 10.42 years of clinical experience (SD = 8.78; range = 1–40 years). All had (at least) a master degree. In addition, 31 (46.3%) had completed a clinical post-doctoral program and 13 (19.4%) were following a clinical post-doctoral program. Therapists followed a workshop provided by one of the authors to inform them about the modular treatment. Most therapists had worked with the protocolized manual before and/or had treated anxiety disorders in children with other standardized CBT programs, and thus had previous experience with the content of the treatment modules. Only 5 therapists (7.5%) reported to have not treated children with anxiety disorders with CBT prior to this study. With regards to treatment adherence, therapists reported which modules were used in each session and whether they used other material/content (not included in the manual). We calculated that only 3% of the treatment time was spent on ‘other material/content’.

### Measures

Treatment effectiveness was measured by the presence (yes/no) of the (primary) anxiety disorder (as measured by the SCID-Junior [35]) and by the amount of anxiety symptoms (as measured by the SCARED-71 [37]). Child predictors were gender and age (as measured by a demographical questionnaire), type of anxiety disorder (as measured by the SCID-junior), and comorbidity (as measured by the SCID-junior and the Brief Problem Monitor [38]). Parent predictors were mothers’ and fathers’ anxiety/depression symptoms (as measured by the anxiety/depression subscale of the Adult Self-report form [39]), and parental involvement in treatment (operationalized as: (1) % of treatment time that parents were present during the sessions as reported by therapists via a treatment rating form, and (2) whether or not the module ‘Parent guidance’ was used in treatment).

#### SCID-Junior

DSM-5 (anxiety) disorders were assessed with the Structured Clinical Interview for DSM-5 Disorders for Children [35]. The SCID-junior integrates parent and child reports to obtain a combined diagnosis. Psychometric properties of the previous version (KID-SCID) have been evaluated [40] and results demonstrated reasonable to good agreement between children’s and parent’s report, and satisfactory to good internal consistencies. In the current study, 10% of the SCID-junior interviews were double coded by an independent rater and interrater agreement based on the presence or absence of the anxiety disorder was high; SCID-junior child report κ = 0.82, SCID-junior parent report κ = 0.72.

##### SCARED-71

The Screen for Child Anxiety Related Emotional Disorders (SCARED-71 [37]) was used to assess child anxiety symptoms. Child and both parent reports were used. The SCARED-71 consists of 71 items which are rated on a three-point scale (0 = (almost) never; 1 = sometimes; 2 = often). In the current study, the total scale of the SCARED-71 was used. The SCARED-71 has good psychometric properties (with high internal consistencies with α’s above > 0.90 for the total score and good construct and discriminant validity; [37, 41]). In the current study, high internal consistency was found for all reporters at all assessments (α > 0.89 for mothers; α > 0.92 for fathers; α > 0.92 for children).

##### BPM

The Brief Problem Monitor [38] was used to assess comorbid symptoms in children. The BPM consists of 19 items which are drawn from the CBCL [42] and can be summed to three subscales (internalizing, externalizing and attention problems) or a total score. The total score at pretest was used in the current study. Each item is rated on a 3-point scale (0 = not true, 1 = somewhat true, 2 = very true) by the mothers and fathers. The BPM has demonstrated good test–retest reliability, validity and internal consistency [42, 43]. In the current study, internal consistency was good (α > 0.80 for mothers; α > 0.77 for fathers).

##### ASR

The subscale anxiety/depression of the Adult Self-Report (ASR) Form for Ages 18–59 [39] was used to measure anxiety/depression symptoms in both parents. The subscale anxiety/depression consists of 18 items that are rated on a 3-point scale (0 = not true, 1 = somewhat or sometimes true, 2 = very true or often true). In the current study, the subscale score obtained at pretest was used. The reliability and validity of the ASR and the anxiety/depression subscale is good [39]. In the current study, internal consistency of the anxiety/depression subscale was also good (α > 0.83 for mothers; α > 0.82 for fathers).

### Data Analyses

Data of 96 children was included in the analyses. Two outcome variables were used: (1) the percentage of children being free from their (primary) anxiety disorder after CBT and (2) the decrease in anxiety problems over time. With respect to the percentages of children being free from their primary anxiety disorder and for the number of children being free from all anxiety disorders, we calculated the percentages based on completer analyses and we calculated the percentages based on intent-to-treat analyses. In the completer analyses, we used the SCID-junior data of the participants who completed mid-, post-, and follow-up assessment. The number of participants who completed SCID-junior assessment was 65 (61.3%), 80 (75.5%), and 88 (83.0%) for respectively mid-, post- and follow-up assessment (note: mid-assessment was completed after five sessions; 20 participants had ≤ 6 sessions and therefore only post-assessment—and not mid-assessment—was completed by these participants). In the intent-to-treat analyses, we used the approach of last observation carried forward to impute missing data about whether an anxiety disorder was present or absent.

For the decrease in anxiety problems over time, we used hierarchical linear model analyses with fixed effects (with maximum likelihood estimation). Repeated measures were nested in respondents (children, mothers and fathers completed the SCARED-71 at pre-, mid-, post- and follow-up assessment), and respondents were nested in families (children, mothers and fathers all reported about the anxiety symptoms of the child). Multilevel analysis uses all available data (including data from families in which not all respondents completed the measurements or in which measures for some assessments in time were missing), so it is not necessary to impute values for missing data. Standardized scores were used in all analyses. Therefore, parameter estimates can be interpreted as Cohen’s *d* (dichotomous predictors) or *r* (continuous predictors). Standardized Z-scores were used to check for outliers (Z-scores > (−)3.29). Outliers were identified for parental involvement and were trimmed when used in analysis. In the first multi-level analysis, we evaluated changes in anxiety symptoms by adding mid-, post- and follow-up to the model as predictors (as contrasted against pretest). In the second set of multi-level analyses, predictors were added for their main effects, and interactions between the predictors and assessments (mid, post and follow-up) were added to evaluate the predictors’ effect on the changes in anxiety over time (during and after treatment). Separate analyses were run for each predictor (child gender, child age, child type of anxiety disorder, child comorbidity, parental anxiety/depression and parent involvement). To control for possible baseline differences, a random intercept model was used. In the third set of multi-level analyses, models were run to examine whether the effect of parental involvement on changes in anxiety symptoms was associated with (1) child age and (2) child comorbidity. To investigate this, three-way interactions (between assessments, parental involvement and child age/ child comorbidity) were added to the models.

## Results

### Treatment Rating Form

The treatment rating form was completed for 84 cases (79%). It was reported by the therapist per child who was present during each session (child, mother, father). In 87% of the cases, at least one parent was involved in treatment in (at least) one session. If parents were involved, both parents were involved in 55% of the cases, mothers only were involved in 36% of the cases, and fathers only were involved in 9% of the cases. In addition, when parents were involved in treatment, there was large heterogeneity in parental presence during sessions; from parents being present at one session (7%) to parents being present every session (100%). Level of parental involvement in the analyses was expressed as the percentage that parents were present in the sessions during the treatment which ranged from 0% (parents were never present in a session) to 100% (parents were always present during the sessions).

Next to the registration of the presence of the child and parents at each session, therapists reported the number of sessions. The mean number of sessions was 9.77 (SD = 4.25; range = 3–27; 6% received 1–5 sessions; 46% received 5–10 sessions, 37% received 10–15 sessions, and 11% received 15–26 sessions). Treatment content varied across children because of the modular approach. The average percentage of treatment time spent on each module was: 14% on Psycho-education, 12% on Cope, 25% on Think, 9% on Feel, 22% on Do, 5% on Parent guidance, and 9% on Relapse prevention (NB. 3% of the treatment time was used on ‘other’; i.e., material not included in the modules). In 95% the module Think (cognitions) was used at least once during treatment, and 76%, 85%, and 84% used at least one time the modules Feel (relaxation and mindfulness), Cope (copings skills), and Do (exposure and/or experiments) respectively. The module Parental guidance (i.e., parenting style, parents’ own dysfunctional cognitions, coparenting, and communication about anxiety) was used at least once during treatment in 44% of the cases. This variable (the use of the module Parental guidance: yes/no) was entered as a predictor as an additional way to examine parental involvement.

### Treatment Outcomes

Table 2 displays the percentages of children being free from their (primary) anxiety disorder at pre-, mid-, post-, and follow-up assessment. Based on completer analyses, 70.2% of the children were free from their primary anxiety disorder at 10 week follow-up, and 62.5% were free of all anxiety disorders. Based on intent to treat analyses, these percentages were 58.5% and 51.9% respectively (see Table 2).

**Table 2:** Treatment outcomes based on % free of (primary) anxiety disorder (AD)

	Completer analyses		Intent to treat analyses (LOCF)	
	Primary AD	Any AD	Primary AD	Any AD
Pre	0.0	0.0	0.0	0.0
Mid	21.5	12.3	13.2	7.5
Post	60.0	47.5	47.2	36.8
Follow-up	70.2	62.5	58.5	51.9

Table 3 provides the results of the multi-level analyses examining changes in anxiety symptoms after treatment. Compared to pretest, anxiety symptoms at mid-, post- and follow-up significantly decreased with an effect size of almost 1 for follow-up (Table 3; see means and SDs across assessments in Supplementary Table 1).

**Table 3:** Decrease in anxiety symptoms (measured by the SCARED-71) since pre-measurement

	Parameter estimate (SE)*	95% CI	t	p
intercept	0.52 (0.09)	0.35 to 0.70	5.87	< .001
Mid	− 0.33 (0.07)	− 0.47 to − 0.18	− 4.57	< .001
Post	− 0.83 (0.08)	− 0.99 to − 0.67	− 10.59	< .001
Follow-up	− 0.98 (0.09)	− 1.16 to − 0.81	− 11.08	< .001

^*^Interpretable as Cohen’s *d* (0.3 = small, 0.5 = medium, 0.8 = large)

### Predictors of Change

Results of the predictor analyses are displayed in Table 4 (note that the main effects of the assessments are not reported for each model separately but the change in anxiety scores across measurements are presented in Table 3). Child predictors (i.e., gender, age, type of anxiety disorder, presence of a comorbid disorder, and comorbid symptoms) were not found to be related to changes in anxiety symptoms (i.e., non-significant interactions between predictors and measurements) with two exceptions: the anxiety scores of children who had a Generalized Anxiety Disorder (GAD) had a steeper decline than the anxiety scores of children with other anxiety disorders (but also note that children with GAD had higher anxiety levels across all assessments; also see Supplementary Table 2), and children who had a Separation Anxiety Disorder (SAD) showed a steeper decline in anxiety scores at post assessment compared to children with other anxiety disorders. Children with comorbid disorders (other than AD; see Table 1) had higher anxiety levels than children without comorbidity, and relatedly, children’s comorbid symptoms (measured with the BPM) were positively associated to their anxiety scores, but comorbidity was not associated with the decrease in anxiety symptoms.

**Table 4:** Results of the multi-level analyses examining predictors of changes

	Parameter estimate (SE)*	95% CI	t	p
Gender (0 = girl; 1 = boy)	− 0.29 (0.18)	− 0.64 to 0.06	− 1.64	0.104
Gender*Mid	− 0.24 (0.16)	− 0.56 to 0.08	− 1.45	0.147
Gender*Post	− 0.16 (0.16)	− 0.47 to 0.15	− 1.03	0.305
Gender*Follow-up	− 0.26 (0.15)	− 0.57 to 0.04	− 1.71	0.088
Age	0.03 (0.09)	− 0.15 to 0.20	0.31	0.757
Age*Mid	0.01 (0.08)	− 0.15 to 0.17	0.15	0.885
Age*Post	0.09 (0.08)	− 0.06 to 0.25	1.16	0.248
Age*Follow-up	0.11 (0.08)	− 0.05 to 0.27	1.40	0.163
Separation AD (0 = no; 1 = yes)	0.26 (0.21)	− 0.15 to 0.68	1.26	0.209
Separation AD*Mid	0.05 (0.18)	− 0.30 to 0.40	0.30	0.766
Separation AD*Post	**− 0.38 (0.18)**	− **0.74 to **− **0.03**	− **2.13**	**0.034**
Separation AD*Follow-up	−0.30 (0.17)	− 0.64 to 0.04	− 1.76	0.080
Social AD (0 = no; 1 = yes)	0.22 (0.18)	− 0.14 to 0.59	1.20	0.230
Social AD*Mid	0.13 (0.17)	− 0.20 to 0.45	0.76	0.447
Social AD*Post	0.14 (0.16)	− 0.18 to 0.45	0.85	0.399
Social AD*Follow-up	−0.05 (0.16)	− 0.35 to 0.26	− 0.29	0.769
**Generalized AD (0 = no; 1 = yes)**	**0.72 (0.18)**	**0.36 to 1.07**	**3.99**	** < .001**
Generalized AD*Mid	0.11 (0.17)	− 0.23 to 0.44	0.62	0.535
Generalized AD*Post	− 0.24 (0.16)	− 0.56 to 0.08	− 1.49	0.135
Generalized AD*Follow-up	− **0.33 (0.16)**	− **0.64 to **− **0.01**	− **2.06**	**0.040**
Specific phobia (0 = no; 1 = yes)	0.25 (0.18)	− 0.11 to 0.62	1.37	0.172
Specific phobia*Mid	− 0.09 (0.17)	− 0.42 to 0.23	− 0.56	0.575
Specific phobia*Post	− 0.30 (0.16)	− 0.61 to 0.01	− 1.91	.057
Specific phobia*Follow-up	− 0.17 (0.16)	− 0.47 to 0.14	− 1.06	.289
**Comorbid disorder (0 = no; 1 = yes)**	**0.45 (0.18)**	**0.09 to 0.81**	**2.48**	**0.014**
Comorbid disorder*Mid	0.00 (0.17)	− 0.33 to 0.22	0.00	0.999
Comorbid disorder*Post	− 0.10 (0.16)	− 0.42 to 0.21	− 0.65	0.514
Comorbid disorder*Follow-up	0.01 (0.16)	− 0.30 to 0.33	0.09	0.926
**Comorbid symptoms (BPM)**	**0.44 (0.09)**	**0.27 to 0.61**	**5.08**	** < 0.001**
Comorbid symptoms*Mid	0.04 (0.08)	− 0.12 to 0.20	0.46	0.647
Comorbid symptoms*Post	− 0.04 (0.08)	− 0.21 to 0.12	− 0.53	0.594
Comorbid symptoms*Follow-up	0.02 (0.08)	− 0.15 to 0.18	0.20	0.845
**Maternal anxiety/depression (ASR)**	**0.20 (0.09)**	**0.01 to 0.39**	**2.12**	**0.036**
Maternal anxiety/depression*Mid	0.04 (0.09)	− 0.14 to 0.22	0.47	0.640
Maternal anxiety/depression*Post	− 0.00 (0.08)	− 0.17 to 0.16	− 0.04	0.967
Maternal anxiety/depression*Follow-up	− 0.04 (0.08)	− 0.20 to 0.12	− 0.51	0.614
Paternal Anxiety/depression (ASR)	0.16 (0.11)	− 0.05 to 0.38	1.48	0.140
Anxiety/depression*Mid	− 0.04 (0.10)	− 0.23 to 0.15	− 0.39	0.700
Anxiety/depression*Post	0.04 (0.09)	− 0.14 to 0.23	0.49	0.629
Anxiety/depression*Follow-up	0.01 (0.09)	− 0.17 to 0.20	0.14	0.893
Parental Involvement	0.04 (0.10)	− 0.17 to 0.24	0.35	0.761
Parental involvement*Mid	− 0.05 (0.09)	− 0.22 to 0.13	− 0.51	0.510
Parental involvement*Post	− 0.15 (0.08)	− 0.31 to 0.02	− 1.75	0.082
Parental involvement*Follow-up	**− 0.20 (0.08)**	**− 0.36 to − 0.03**	**− 2.36**	**0.019**
Parental module used in treatment	0.13 (0.20)	− 0.27 to 0.52	0.65	.519
Parental module*Mid	− 0.05 (0.16)	− 0.37 to 0.27	− 0.30	.767
Parental module*Post	− 0.30 (0.17)	− 0.65 to 0.04	− 1.74	.087
Parental module*Follow-up	− 0.09 (0.19)	− 0.46 to 0.29	− 0.46	.647

Bold values indicate significanace of p value (*p* < 0.05)

*AD* anxiety disorder (measured by the SCID-junior), *ASR* Adult Self-Report Form, *BPM* Brief Problem Monitor, *Comorbid disorder* disorder other than AD (see Table 1), *Parental Involvement* number of the sessions in which parents were present/number of total sessions * 100; SCARED-71 = Screen for Child Anxiety and Related Emotional Disorders

Maternal (but not paternal) anxiety/depression scores were found to be related to child anxiety scores in the direction that higher anxiety/depression scores in mothers were associated with higher anxiety levels in their children. However, maternal as well as paternal anxiety/depression scores were not related to changes in child anxiety symptoms over time. Higher level of parental involvement (expressed as the percentage of treatment time that parents were present during the sessions) in treatment was related to a higher decrease in anxiety scores at post assessment and follow-up. However, the use of the module Parental guidance (yes/no) in treatment was not related to the decrease in child anxiety symptom scores.

Three-way interactions were examined next. No significant three-way interactions between assessments, parental involvement and child age were found, however, a significant three-way interaction between assessment (follow-up), child comorbidity (yes/no comorbid non-anxiety disorder based on SCID diagnosis, see Table 1) and parental involvement (expressed as the percentage of treatment time that parents were present during the sessions) was found. Post hoc analyses revealed that at follow-up, the level of parental involvement was not associated with greater changes in anxiety scores for children without comorbid disorders (*parameter estimate* = 0.07, *p* = 0.517), but for children with comorbid disorders higher parental involvement in treatment was associated with less anxiety symptoms at follow-up (*parameter estimate* = − 0.35, *p* = 0.004).

## Discussion

The current study examined the outcomes of a modular CBT for childhood ADs in a community clinics sample, as well as predictors of change. Main findings can be summarized as follows: (1) the modular therapy with an average amount of almost 10 sessions appears effective, with a within group effect size of almost 1 and 59% (intent-to-treat analysis) to 70% (completer analysis) of the children being free from their primary anxiety disorder at 10 weeks follow up; and (2) treatment changes were not predicted by child age, gender, comorbidity, or parental anxiety/depression symptoms; however, having a generalized anxiety disorder (GAD) was associated with a steeper decrease in anxiety at follow-up and having a separation anxiety disorder was associated with a steeper decrease in anxiety at post-assessment. In addition, higher parental involvement (expressed as the percentage of treatment time that parents were present during the sessions) was associated with higher decreases in anxiety at follow-up, but only for children with comorbid disorders. However, the use of the parental module in treatment was not related to the decrease in anxiety symptom scores.

The clinical outcomes of the modular CBT in the current study were found to be in line with previous reported effects of non-modular CBT for child anxiety disorders (e.g., see meta-analyses [27, 28, 44, 45]. This finding is promising considering the average number of sessions being (only) 10 sessions in the current study – which is quite low compared to non-modularized CBT protocols (a mean of 13 sessions was reported in a review including 41 treatment studies [46]), and the clinical nature of the study. That is, our study was performed in usual clinical practice with only a few inclusion criteria and no exclusion criteria for participants, therapists’ education level, inter- or supervision, or mental health care centers. Although we cannot draw any conclusions regarding the effects of modular CBT above and beyond standardized CBT based on this study, it seems at least plausible that using a modular approach is beneficial (and potentially cost-effective) for children with ADs. However, RCT’s comparing costs and effects of modular versus standardized treatment are needed.

In agreement with most studies examining non-modularized CBT, we did not find evidence for child age or gender predicting treatment outcomes [48]. We did find some evidence that type of AD is associated with treatment changes. That is, anxiety scores of children with separation anxiety disorder or GAD showed a steeper decline compared to children with other ADs which is in line with previous studies where these ADs have been linked to better treatment effectiveness as well [12, 13, 49]. However, baseline anxiety scores for children with generalized AD were found to be higher than children without generalized AD which might have led to more room for improvement, but also note that higher anxiety scores for children with generalized AD were found across all assessments including follow-up. Thus, although these children showed a steeper delice in anxiety symptoms, their scores at follow-up were still higher compared to anxiety scores of children without generalized AD. We did not find support for that children with social AD benefit less from treatment as was found in previous studies using non-modular CBT [13, 49]. An explanation could be the potential differences in content of the modular treatment compared to other (non-modular) CBT. That is, not only could therapists decide how many sessions were spent on what content (modules) and in which order, they were also able to choose modules including task concentration and mindfulness, which helps the child become less self-focused (e.g., heightened self-focused attention is regarded as an important maintaining mechanism in social anxiety in adults as well as in children [50–52]).

With regards to parental involvement, we found that higher parental involvement (expressed as the amount of time that parents were present during sessions) was associated with a higher decrease in anxiety symptoms but additional analyses showed that this was only for children with comorbid disorders (most common comorbid disorders in this sample were ADHD, insomnia and mood disorders, see Table 1). Possibly, children with comorbid disorders benefit from parental involvement as they need not only anxiety treatment, but also need to deal with their comorbid problems. For example, a child with ADHD may have problems with planning exposures, a child with depression may need to be stimulated (more) by its parents to actively engage in exposures, and a child with ASD may need its parents to translate exposures to daily practice. It is important to note here that parental involvement was not a random factor, but it was deliberately chosen by therapists (and clients). Thus, the result may reflect that therapists have chosen wisely whether or not to involve parents, and at what time (e.g., involve parents on forehand, or when treatment stagnated). A further complication is that we do not know the nature of parental involvement in the treatment sessions: e.g., involving parents as collaborators or as co-clients, targeting parenting behaviors such as controlling or autonomy-granting, or supporting parents to do exposures with their child at home. With regards to this issue, we did not find evidence that the use of the module Parent guidance in treatment was related to the decrease in anxiety symptom scores. Issues addressed in this module include: parenting style (overprotection, modelling, autonomy granting behaviors), parents’ own dysfunctional cognitions, coparenting, and communication about anxiety. This suggests that these issues are not an essential type of parental involvement that is related to treatment success. We could speculate that based on previous research [24], contingency management (which is part of the module exposure) or transfer of control might be important ingredients for parental involvement, or that parents’ presence during the sessions is important for the generalization of learnt skills to daily settings for children with comorbidity. However, we did not measure these components explicitly in the current study and therefore we are not able to draw any conclusions about the type of parental involvement. Future studies (with a more experimental design) may be used to study the effects of different components of parental involvement in childhood anxiety treatment. This may be specifically relevant for children with comorbid disorders, as previous research findings demonstrate that parental involvement for children with comorbid ASD- or ADHD-problems is more important [16, 31, 53].

The main strength of this study is that it evaluated treatment outcomes, and its predictors, in a large sample of clinically referred children who were treated for their anxiety problems in community clinical practices not specifically specialized in treating anxiety disorders. In addition, therapists were unselected, coming from multiple treatment centers and had various educational backgrounds, and were not supervised by the research team during the delivery of treatment. However, some limitations also need to be addressed. First, there was no comparison group (receiving non-modular CBT), so there is no way of knowing whether modular CBT would outperform manualized CBT for childhood ADs in clinical practice. Second, no random assignment of parental involvement was made, and we also do not know in what way parents were involved. Although such an approach contributes to the external validity of the study, it is difficult to draw firm conclusions as to whether and how parental involvement contributes to treatment outcomes. Third, parental anxiety/depression symptoms were based on a self-reported questionnaire, and there was no measure for parental anxiety disorder. In addition, important to note is that parental anxiety/depression can change during the course of child treatment, and it may be that the change—rather than the anxiety/depression level at baseline—is related to changes in child anxiety. Finally, a longer-term follow-up assessment is lacking, which is unfortunate as it has been suggested that parental involvement leads to more remission in the (long-term) period following treatment [54].

To conclude, the study results suggest that modular CBT can be effective for the treatment of childhood ADs in community mental health care centers as recovery rates of ADs and effect sizes in this study seem comparable to those of previous (research therapy) studies. The fact that parental involvement was not randomized in the current study hampers more definite conclusions regarding the role of parental involvement for children with comorbidity. However, the study findings do suggest that it appears beneficial to involve parents when the child has comorbid problems (most common comorbid disorders were ADHD, insomnia, and mood disorders).

## Summary

Cognitive Behavioral Therapy (CBT) is effective for childhood anxiety disorders. However, around one third of the children are not (fully) recovered from their (primary) anxiety disorders after CBT. Most of the previous (research therapy) studies used a structured treatment protocol with a limited number of sessions and used a selected sample of participants who might be less representative for clinical community settings. In addition, in clinical practice, treatment is often adjusted to the needs of the individual child, for example by adopting a more flexible treatment approach (e.g., adjusting the number of sessions or adjusting treatment content). One way to use a more flexible approach is to use a modular format. Therefore, the current study examined the clinical outcomes of a modular CBT for children with anxiety disorders, and examined predictors of change. We found that 59% of the children (intent-to-treat analyses) to 70% of the children (completer analysis) were free of their primary AD at follow-up, and a large decrease in anxiety symptoms was found. The percentages of children being free from their (primary) anxiety disorder seem comparable to other studies evaluating standard (non-modular) CBT, while the average number of sessions was (only) 10. Regarding predictors of change, the current study found that children with Generalized AD and children with separation anxiety disorders displayed steeper declines in their anxiety levels than children with other ADs. Findings also suggests that involvement of parents in treatment is beneficial when the child has comorbid problems.

### Supplementary Information

Below is the link to the electronic supplementary material.

Supplementary file1 (DOCX 19 KB)

## Data Availability

The data that support the findings of this study are available from the corresponding author, upon reasonable request.
