# Supplementary material for: Modular CBT for Childhood Anxiety Disorders: Evaluating Clinical Outcomes and its Predictors
Source: Child Psychiatry Hum Dev. 2022 Oct 3;55(3):790–801. doi: 10.1007/s10578-022-01437-1 (PMC11061043; doi:10.1007/s10578-022-01437-1)
Supplement: Supplementary file 1 — Supplementary file1 (DOCX 19 KB) [file 10578_2022_1437_MOESM1_ESM.docx]

Supplementary Table 1. Means (SD) for the study variables

|  | **Child** | **Mother** | **Father** |
| --- | --- | --- | --- |
| Anxiety Symptoms (SCARED-71) |  |  |  |
| *Pre* | 51.24 (20.12) | 48.06 (17.21) | 41.58 (18.34) |
| *Mid* | 47.89 (21.74) | 42.23 (17.24) | 36.52 (17.47) |
| *Post* | 37.78 (19.15) | 31.42 (17.24) | 29.90 (17.49) |
| *Follow up* | 34.40 (19.95) | 28.45 (18.53) | 23.23 (18.17) |
| Comorbid symptoms (BPM) |  | 14.08 (6.02) | 12.65 (5.44) |
| Parental Anxiety/Depression (ASR) |  | 5.29 (4.12) | 4.23 (3.70) |
| Parental Involvement | 11.96 (31.02) |  |  |

*Note.* ASR = Adult Self-Report Form; BPM = Brief Problem Monitor; Parental Involvement = number of the sessions in which parents were present / number of total sessions * 100; SCARED-71 = Screen for Child Anxiety and Related Emotional Disorders

Supplementary Table 2. Means (SD) for the children with and without a particular type of anxiety disorder (child, mother and father report aggregated).

|  |  | Children with type of AD mentioned in first column | | Children without type of AD mentioned in first column | |
| --- | --- | --- | --- | --- | --- |
|  |  | M | SD | M | SD |
| Generalized Anxiety disorder | Pre | 51.81 | 14.47 | 38.84 | 16.71 |
|  | Mid | 45.72 | 17.99 | 39.60 | 16.08 |
|  | Post | 36.36 | 15.03 | 24.97 | 11.95 |
|  | Follow-up | 32.24 | 17.15 | 23.22 | 15.17 |
| Social anxiety disorder | Pre | 48.60 | 17.18 | 44.81 | 15.61 |
|  | Mid | 44.32 | 18.23 | 38.16 | 14.52 |
|  | Post | 34.44 | 14.07 | 28.69 | 15.54 |
|  | Follow-up | 27.28 | 17.48 | 30.17 | 16.52 |
| Separation anxiety disorder | Pre | 50.86 | 14.40 | 45.47 | 17.10 |
|  | Mid | 47.66 | 15.68 | 30.45 | 12.83 |
|  | Post | 30.77 | 16.51 | 32.31 | 14.46 |
|  | Follow-up | 29.05 | 17.62 | 28.36 | 15.56 |
| Specific phobia | Pre | 49.39 | 16.76 | 44.54 | 16.10 |
|  | Mid | 42.68 | 17.96 | 40.06 | 15.41 |
|  | Post | 31.29 | 15.03 | 32.57 | 14.96 |
|  | Follow-up | 29.63 | 17.00 | 27.97 | 17.00 |
